# Psychosocial empowerment following orthognathic surgery for patients born with cleft lip and/or palate

**DOI:** 10.1080/17482631.2026.2674347

**Published:** 2026-05-15

**Authors:** Joakim Lundberg, Mats Sjöström, Jenny Molin

**Affiliations:** a Department of Odontology, Oral and Maxillofacial Surgery, Umeå University, Umeå, Sweden; b Department of Nursing and Department of Clinical Science, Division of Psychiatry, Umeå University, Umeå, Sweden

**Keywords:** Cleft lip and palate, orthognathic surgery, qualitative content analysis, psychosocial well-being, Patients’ experiences, self-confidence

## Abstract

**Background:**

Individuals born with cleft lip and/or palate (CLP) may experience functional, esthetic and psychosocial challenges. While orthognathic surgery is performed to correct jaw discrepancies, knowledge about patients’ experiences of this treatment remains limited, as previous research has largely relied on quantitative quality-of-life measures.

**Objective:**

This study aimed to illuminate the experiences of individuals with CLP undergoing orthognathic surgery.

**Methods:**

An inductive qualitative design was used. Individual semi-structured interviews were conducted with 16 adults treated with orthognathic surgery. Data were analyzed using qualitative content analysis.

**Results:**

One overarching theme, *Emerging empowered through a life-changing challenge,* described surgery as, for many, a transformative process extending beyond functional and esthetic correction. Three themes described participants’ experiences: being highly motivated for change, overcoming tough setbacks through a stable backing system, and enhancing self-confidence. While many participants described increased confidence and social ease over time, empowerment was neither universal nor immediate, with some reported neutral or context-dependent psychosocial outcomes.

**Conclusions:**

Orthognathic surgery may be experienced as a transformative process within a lifelong CLP trajectory, with potential to foster psychosocial empowerment and renewed self-confidence. These findings highlight both the transformative possibilities and the contextual variability of surgical outcomes in person-centered cleft care.

## Introduction

Cleft, lip and/or palate (CLP) is one of the most common congenital conditions worldwide, with a prevalence of 1 in 700–1,500 liveborn children depending on the different parts of the world (Kloukos et al., 2018; Mossey et al., 2009). Orofacial clefts can involve different anatomic structures and be divided into the following subcategories: cleft lip (CL); cleft lip and palate (CLP); and cleft palate (CP) (Mossey et al., 2009). The clinical features of CLP differ between patients born with a cleft. As a consequence of the cleft, the teeth, jaw and facial appearance can be affected, as well as functional aspects such as speech and feeding (Berkowitz, 2013). A multidisciplinary team is involved in the case of the affected patient from birth to 19 years of age (Peterson, 2022).

Patients with CLP have a higher risk of maxillary hypoplasia due to scar tissue formed as a result of previous palatoplasty (Shi & Losee, 2015). The maxillary hypoplasia leads to altered jaw relations. To correct these jaw discrepancies, orthognathic surgery is used to reposition the maxilla when orthodontic treatment alone proves insufficient (Ross, 1987). The repositioning of the maxilla can be done by either conventional orthognathic surgery (CO) or distraction osteogenesis (DO) (Kloukos et al., 2018). DO can be used for patients with CLP, so as to limit skeletal relapse due to the presence of scar tissue in the maxilla from previous surgeries (Chua et al., 2010).

Individuals with CLP may experience challenges related to facial appearance, social interactions and psychosocial well-being, including lower satisfaction with appearance and reduced Quality of Life (QoL) compared with unaffected populations (Al-Namankany & Alhubaishi, 2018; Lorot-Marchand et al., 2015; Mani et al., 2010; Marcusson et al., 2002; Peroz et al., 2024). Orthognathic surgery has been associated with improved psychological well-being and life satisfaction in the longer term (Chua et al., 2012; Feitosa et al., 2022). However, these outcomes have primarily been explored using standardised patient-reported outcomes measures, which may overlook aspects of experience meaningful to patients but not captured by predefined domains (Pyo et al., 2023).

Applying qualitative methods has been shown to provide rich descriptions of experiences and deepen understanding of health phenomena, offering insights that can complement quantitative QoL measures and contribute to more comprehensive, person-centred evaluations (Alansari et al., 2014; Oosterkamp et al., 2007; Renjith et al., 2021).

The aim of this study is to illuminate the experiences of individuals with CLP undergoing orthognathic surgery.

## Material and methods

### Design

The study was conducted within an interpretive epistemological framework, assuming that experiences of orthognathic surgery are subjectively constructed and shaped by social and contextual factors. Knowledge was understood as co-created in the interaction between interviewer and participant. The study employed an inductive qualitative design using individual semi-structured interviews and inductive qualitative content analysis (Graneheim & Lundman, 2004; Graneheim et al., 2017).

This interpretivist stance guided the analytic focus on participants’ meaning-making processes including experiences that could involve psychosocial meanings following orthognathic surgery, rather than on functional or biomedical outcomes. The analysis therefore aimed to interpret how experiences were constructed and narrated over time, rather than to measure prevalence or infer causality.

### Context

In Sweden, children born with CLP are referred to a multidisciplinary CLP team immediately after birth. They are examined and receive an individualised treatment plan at one of the six regional CLP centres. Umeå University Hospital is responsible for children with CLP residing in the four northern regions of Sweden. The Swedish CLP care system is publicly funded and nationally standardised, aiming to provide equal access to long-term multidisciplinary follow-up across regions.

The Swedish CLP teams treat children with CLP according to a standard treatment protocol for the specific cleft, from birth to 19 years of age with regular follow-up appointments during the period of childhood. If extended care is needed, the follow-ups continue after 19 years of age. Orthognathic surgery or secondary corrections are examples of treatments that may be performed after 19 years of age. The CLP teams consist of a group of healthcare professionals, including oral and maxillofacial surgeons, plastic surgeons, orthodontists, speech pathologists, nurses and ear nose and throat specialists (ENT).

Author JL is a PhD student and a medical doctor in residency training, with MS as the main supervisor. MS is an oral and maxillofacial surgeon and a member of the CLP team at Umeå University Hospital. Both have prior experience in quantitative research on patients with CLP. Author JM is a mental health nurse with experience in qualitative research, contributing perspectives from outside the CLP field.

### Participants

Participants were recruited from the regional CLP centre at Umeå University Hospital using consecutive inclusion of all eligible participants identified through clinical records. All individuals who met the inclusion criteria during the recruitment period were invited by letter, followed by telephone contact. Thus, no purposive selection was undertaken; rather, variation in age, sex, surgical technique and postoperative interval emerged within the final sample through inclusion of all consenting patients.

The inclusion criterion was individuals treated with orthognathic surgery to normalise basal discrepancies between the maxilla and mandible at Umeå University Hospital. In total, 22 patients with CLP were eligible for this study and 16 (73%) participants (12 men and 4 women) agreed to be included in the study (mean age, 31.0 years; range, 21.0–41.6 years). At the time of surgery, the mean age was 20.7 years (range, 14.8–26.1 years). The mean elapsed time between the surgery and the interviews was 8.9 years (range, 0.3–19.8 years).

Eleven participants received CO and five underwent DO. Prior to surgery, 13 participants received two-stage palatoplasty while 3 participants received one-stage palatoplasty. Three participants underwent velopharyngeal closure prior to orthognathic treatment. Four participants were adopted and, therefore, not treated by the Umeå CLP team from birth but came in contact with the CLP team following arrival in Sweden. All the remaining participants received care from birth.

The study did not aim to compare surgical techniques (CO vs DO) or postoperative intervals, but rather to explore experiential meaning across varied clinical trajectories. However, we acknowledge that such heterogeneity may influence how experiences are recalled or articulated. These dimensions were therefore considered analytically as part of the experiential context rather than as variables for comparison. This approach allowed for identification of both shared and divergent patterns while maintaining focus on meaning-making processes.

### Data collection and procedure

The interviews were conducted either in a conference room at Umeå University Hospital in a non-clinical setting or by telephone, where the participants were at home during the interview. While interviews were conducted both face-to-face and by telephone, we acknowledge that differences in modality may influence data richness. Telephone interviews may limit access to non-verbal cues but can also facilitate openness when discussing sensitive experiences. These potential differences were considered during analysis and interpretation. Telephone interviews were considered appropriate to facilitate participation among individuals living in geographically distant regions.

Before the interviews were conducted, all participants were informed about the study and the possibility to withdraw from the study at any time without giving any reason. All participants provided written consent before participating in the study.

All the interviews were performed in Swedish, the participants’ native tongue, using a semi-structured interview guide. Translation to English was performed during the subtheme phase of analysis. Translation was conducted by the first author and reviewed in collaboration with co-authors to ensure that meanings were preserved. Particular attention was paid to maintaining conceptual equivalence rather than literal translation. Formal back-translation was not undertaken, as coding and early-stage analysis were conducted in the original language and translation occurred only at the subtheme phase.

After the participants signed the consent form, audio-recorded interviews were conducted by the first author, who did not have any knowledge regarding the participants prior to this study.

A pilot interview was conducted, and the results were discussed with the last author, to assess the quality of the information and to adjust the interview guide to maximise the quality of the information collected in the subsequent interviews. No adjustments were made to the interview guide. The data from the pilot interview was included in this study. The guide covered open-ended questions regarding experiences related to the orthognathic surgical procedure. The interviews lasted on average 39 minutes (range, 28–58 minutes). All the interviews were transcribed verbatim and then cross-checked with the audio files.

Data collection continued until sufficient information power was achieved, in accordance with Malterud et al. (2^016^). Information power was evaluated continuously based on (1) the narrow and specific aim of exploring psychosocial experiences following orthognathic surgery, (2) the specificity of the sample (individuals with CLP undergoing similar treatment trajectories), (3) the quality and depth of the interview dialogue, (4) the application of established theoretical perspectives within health and psychosocial research, and (5) the chosen analytical strategy.

The focused exploratory aim of illuminating experiences of orthognathic surgery among individuals with CLP, combined with rich dialogue, supported the adequacy of the sample in relation to the concept of information power. During the analytical process, the data were considered sufficiently rich and nuanced to allow for meaningful pattern identification and interpretation.

### Data analysis

The data were subjected to inductive qualitative content analysis with both manifest and latent interpretation (Graneheim & Lundman, 2004; Graneheim et al., 2017). This approach was chosen as it allows for systematic identification of patterns across participants while preserving the contextual meaning of participants’ narratives. Given the study’s aim to explore both explicit experiences and underlying meanings related to surgical treatment, qualitative content analysis was considered suitable. Data was managed using spreadsheet software, Microsoft Excel 365. The analysis started with division of the text into meaning units, which were then condensed. Each condensation was labelled with a code. The codes were kept close to the text. Codes were clustered and thereafter, clusters were abstracted and interpreted into subthemes. Subthemes with similar content were clustered, abstracted and interpreted into themes. An example of the analytical process from meaning unit to code, subtheme, and theme has been included (Table I). The first author performed the initial coding. The coding structure, subthemes and themes were then discussed in repeated meetings with the co-authors. All authors reviewed and critically reflected upon the coding process. Disagreements were resolved through discussion until consensus was reached. To enhance trustworthiness, coding decisions were continuously discussed among authors, and preliminary interpretations were revisited in relation to the original data.

**Table I.** Example of the analytical process from meaning unit to code, subtheme and theme.

Meaning unit	Code	Subtheme	Theme
The apperance was a big factor, the face profile was horrible	Appearance big factor face profile horrible	Taking the decision based on physical and aesthetic issues	Being highly motivated for change
There was a period of severe pain in the face	Period of severe pain	Enduring the aftermath	Overcoming tough setbacks through a stable backing system
Social life changed, people started to initiate contact	Social life changed people initiate contact	Experiencing improved psychosocial well-being	Enhancing self-confidence

### Reflexivity

Reflexivity was considered throughout the research process. The second author’s role as an oral and maxillofacial surgeon within a CLP team provided clinical insight but also carried a risk of pre-understanding influencing interpretation. To mitigate this, the first author conducted all interviews independently and had no prior clinical relationship with participants. Analytical discussions were conducted collaboratively with the third author, who has no clinical background in CLP care, thereby contributing an external perspective. We also acknowledge that the involvement of a clinician-researcher may have influenced which aspects were perceived as clinically salient during interpretation. This was actively discussed to remain attentive to participants’ own framings and to avoid privileging biomedical perspectives.

### Ethical considerations

The study was carried out according to the ethical standards of the Helsinki Declaration and was approved by the Regional Ethical Review Board in Umeå (Dnr: 2016-205-31M). All participants were informed about the study and gave their written consent to participate. All patient data were anonymized.

To preserve anonymity, the interviews were conducted without anyone else present besides the participant and first author, and confidentiality regarding the information and the audio files was followed throughout the study. The participants were also reminded that they were free to withdraw at any moment during the study.

## Results

The analysis resulted in one overarching main theme, *Emerging empowered through a life-changing challenge*, which captures how orthognathic surgery was experienced as extending beyond functional and aesthetic correction, influencing participants’ self-perception and social participation in varied ways. The participants’ narrative described a journey from long-standing psychosocial burden and strong motivation for change, through physically and emotionally demanding postoperative challenges, to enhanced self-confidence and improved quality of life. Three themes were identified: *Being highly motivated for change, overcoming tough setbacks through a stable backing system* and *enhancing self-confidence*. Each theme comprised two to three subthemes that together illuminate different phases of the participants’ experience before, during and after surgery. An overview of the results is presented in Table II.

**Table II.** Overview of the results.

Subtheme	Theme	Main theme
Taking the decision based on physical and aesthetic issues Dealing with challenging emotions	Being highly motivated for change	Emerging empowered through a life-changing challenge
Enduring the aftermath Recovering through support and continuity of care	Overcoming tough setbacks through stable backing system	
Experiencing improved psychosocial well-being Neutral psychosocial impact Finding the drive to change	Enhancing self-confidence	

### Emerging empowered through a life-changing challenge

Across the interviews, participants described how living with CLP had profoundly affected their self-worth, social participation and sense of identity. Prior to surgery, many felt withdrawn, avoided social interactions and perceived themselves as defined by their facial difference. These experiences generated a strong desire for change. Although the surgical process involved substantial physical and emotional hardship, participants described how support from others and gradual improvements after surgery enabled them to reclaim confidence, re-engage socially and develop a renewed sense of self-worth. The main theme reflects a dynamic and sometimes ambivalent process in which participants renegotiated their sense of self and social agency through embodied surgical change. While many described increased confidence and participation, this transformation was neither immediate nor uniform, but unfolded through periods of uncertainty, dependence and adjustment.

### Being highly motivated for change

This theme captures the factors that contribute to participants’ strong motivation to undergo orthognathic surgery. The motivation was rooted both in dissatisfaction with appearance and function and in emotionally challenging experiences related to self-esteem, social exposure and anticipation of surgery.

#### Taking the decision based on physical and aesthetic issues

Participants described dissatisfaction with their appearance, particularly related to an underbite and altered jaw relations, which made smiling in public and showing their teeth difficult. Some reported experiences or bullying during childhood and adolescence, which negatively affected their self-esteem and contributed to persistent feelings of being different. Chewing difficulties varied, ranging from minor inconvenience to marked avoidance of certain foods, such as meat of hard textures. Several participants described feelings of shame related to how they chewed, sometimes choosing to eat alone or hide their mouth when eating in public. Functional pain from the jaws was also mentioned as a motivating factor for surgery.

*“… I was tired of my underbite. I had a strong complex about smiling and showing my teeth because my smile didn’t look good. I also wanted to chew better, I couldn't chew meat for example and I avoided certain foods. And my appearance was very masculine due to my underbite, and I wanted a more feminine appearance.”*—P10

While most participants described the decision to undergo surgery as their own, some experienced the decision as being strongly influenced by dental professionals.

#### Dealing with challenging emotions

Emotional responses prior to surgery included nervousness, anxiety and ambivalence. Some participants managed their worries by sharing concerns with family members, others chose not to in order to protect their loved ones. Several expressed a desire to speak with individuals who had previously undergone surgery, seeking practical information and reassurance. For some, receiving a surgery date generated excitement and renewed motivation, as the end of long-term treatment appeared within reach.

One participant described how negative experiences with anaesthesia heightened preoperative anxiety, but how responsive staff helped restore a sense of control.

*“I said: You will be able to sedate me, but I will not take any medication before I’m ready. We talked for a while and then I gave them my arm. Couldn’t have had better treatment! Had a very good experience from it.”*—P16

### Overcoming tough setbacks through a stable backing system

This theme describes the physically and emotionally demanding postoperative period and the factors that enabled participants to cope with these challenges. The subthemes illustrate both the hardships encountered after surgery and the importance of support and continuity of care in facilitating recovery.

#### Enduring the aftermath

The early postoperative period was described as particularly challenging. Participants reported pronounced swelling, fatigue and sensory disturbances, which often resulted in temporary social withdrawal and reduced activity levels. Liquid diets linked with reduced appetite and altered taste after surgery were common among participants, and some of them described adding salt and pepper to yoghurt to enhance the taste. Adjusting to new jaw relationships was frequently accompanied by tension-related pain. Experiences of pain varied widely, ranging from manageable discomfort to what some described as the worst pain they had experienced.

“*I wasn’t a fun boyfriend at the time. I sat home all day playing video games and felt depressed during the healing process.”*—P13

For some participants, this period also triggered doubts about whether the anticipated benefits would outweigh the burden of recovery. The vulnerability experienced during this phase contrasted sharply with preoperative expectations of positive change.

For participants who underwent DO, the prolonged treatment phase was perceived as particularly demanding, although these challenges were generally understood as temporary and part of a necessary process.

#### Recovering through support and continuity of care

Support from loved ones was described as crucial throughout the recovery process. Emotional reassurance, practical help with daily activities and encouragement regarding surgical outcomes contributed to a sense of safety, motivation and enhanced the participants’ self-esteem during a vulnerable phase in their rehabilitation.

*“They were always there and supported me…. Their positive comments on surgical results strengthened me.”*—P6

Participants also emphasised the importance of respectful, responsive healthcare staff. Adequate pre- and postoperative information helped participants feel prepared and reassured, whereas insufficient information contributed to distress and uncertainty. Follow-up appointments were described as calming and essential for confirming that recovery was progressing as expected. The effects on engaging in education due to surgery varied among the participants, with those who studied remotely being least affected. Some fell behind in school assignments but caught up with schoolmates in a few weeks. For some participants, this involved experiencing high-level stress during the catch-up-phase.

### Enhancing self-confidence

This theme reflects the longer-term outcomes of surgery, characterised mostly by improved quality of life and increased self-confidence. The subthemes describe how functional and aesthetic improvements are translated into broader changes in self-perception and behaviour. Although most participants described positive changes, some also expressed a need to adjust to their altered appearance and social responses, suggesting that even positive transformation required psychological integration over time.

#### Experiencing improved psychosocial well-being

Participants described orthognathic surgery as life-changing, with restored facial aesthetics and improved jaw function contributing to increased confidence. Many highlighted the impact of receiving compliments about their appearance, which was a new and affirming experience. Participants who had previously been socially withdrawn described becoming more outgoing and confident in social interactions, leading to a substantial improvement in perceived quality of life.

*“After the surgery I became much more socially outgoing, and it completely changed my life”*—P15

#### Neutral psychosocial impact

Although many participants described positive experiences regarding psychosocial impact of orthognathic surgery, this was not universal.

*“I don’t believe the surgery made any difference…. I wasn’t affected by it, neither positive or negative.”*—P2

For some participants, the surgery was described as leading to functional changes, but without noticeable changes in self-perception or other psychosocial aspects of life. They expressed that, although their appearance had changed, the surgery had not influenced how they viewed themselves or how they experienced their social situation. These accounts served as important disconfirming cases in the analysis.

#### Finding the drive to change

Improved jaw relations enabled participants to chew and eat without restriction, which was experienced as a meaningful functional gain. Beyond these direct effects, participants described a broader motivational shift after surgery. Some reported aspirations for additional corrective procedures, while others described renewed motivation to improve general health through lifestyle changes such as exercise and healthier eating habits. Increased self-esteem also encouraged changes in appearance and self-expression that had previously felt unattainable.

*“When I came back to school, I wanted to do something completely different for me, so I dyed my hair. It was great to see my classmates’ reactions.”*—P10

Together, these experiences illustrate how orthognathic surgery contributed not only to functional and aesthetic improvements but also to enhanced self-confidence, agency and engagement in life.

## Discussion

The purpose of this study was to illuminate the experiences of participants with CLP who were undergoing treatment with orthognathic surgery. Participants’ narratives illustrated a gradual process of psychosocial renegotiation, moving from preoperative vulnerability to varying degrees of increased confidence and social participation.

Beyond functional and aesthetic correction, the findings illustrate orthognathic surgery as a potentially transformative process that reshapes participants' sense of self, social positioning and agency. The overarching theme, *Emerging empowered through a life-changing challenge*, can be understood as a gradual reconfiguration of identity, in which long-standing experiences of vulnerability were renegotiated through bodily change, social feedback and increased participation in everyday life. Previous research within the field of visible difference has similarly highlighted how surgical and medical interventions may involve processes of identity renegotiation rather than solely functional change (Harcourt & Rumsey, 2005; Stock et al., 2015). Participants’ narratives suggested that changes in facial appearance were intertwined with changes in self-perception, confidence and social participation.

Importantly, not all participants described psychosocial gains following surgery. For some, the intervention resulted primarily in functional or aesthetic change without substantial transformation of self-concept or social participation. These accounts complicate a purely transformative narrative and suggest that orthognathic surgery did not uniformly lead to broader psychosocial change. Previous studies within CLP populations have similarly demonstrated heterogeneity in psychosocial adjustment and body image outcomes (Stock & Feragen, 2016).

In addition, participants’ increased self-agency over time may partly reflect age-related psychological development rather than surgery alone. Orthognathic treatment is typically undertaken during late adolescence or young adulthood, a life phase characterised by identity consolidation and increased autonomy. Previous research has demonstrated that the psychosocial impact of CLP varies across the lifespan, with social rejection and isolation reported more frequently during school years (Nicholls et al., 2019). Future research comparing individuals who decline surgery with those who proceed may help disentangle surgical effects from developmental trajectories.

The strong motivation for change described by the participants may be understood not only as dissatisfaction with appearance or function, but as a response to cumulative psychosocial strain over time. Experiences of social exposure, bullying and persistent self-monitoring appeared to shape a desire for change that extended beyond the physical correction of the jaws, reflecting a wish to alter how the self was perceived and engaged in social contexts. For individuals with CLP, orthognathic surgery does not represent an isolated intervention, but rather a pivotal moment within a lifelong trajectory of treatment and adaptation.

The participants’ accounts of cumulative psychosocial strain align with broader research indicating elevated mental health vulnerability among individuals with CLP compared to normative populations (Mani et al., 2010; Wang et al., 2024). However, while quantitative studies describe prevalence patterns, the present findings illuminate how such vulnerabilities are experienced, internalised, and negotiated over time. Experiences of stigma and negative self-image have also been linked to increased willingness to undergo corrective surgery (Alansari et al., 2014). Within this context, the strong motivation described by participants in the present study may reflect both cumulative psychosocial strain and hope for improved self-perception and functional well-being.

Participants described significant postoperative challenges, including pain, swelling and temporary sensory disturbances. Previous research has demonstrated that early postoperative pain may negatively affect oral health-related quality of life (Tuk et al., 2022). Differences between DO and CO have also been reported in short-term psychosocial outcomes, although these differences diminish over time (Chua et al., 2012). As the present study aimed to explore meaning-making rather than comparative outcomes, surgical techniques were not analysed separately.

Several participants emphasised the importance of stable support systems and adequate preoperative information in reducing distress and aligning expectations. Previous research has shown that unrealistic expectations regarding additional surgery may lead to postoperative dissatisfaction (Alansari et al., 2014; Stock et al., 2016). Qualitative findings by Hamlet and Harcourt (2015) further suggest that psychosocial support may be beneficial beyond the immediate treatment period (Hamlet & Harcourt, 2015). Together, these findings support consideration of structured psychological assessment and follow-up within the multidisciplinary cleft care.

Participants described initiating interactions, expressing themselves more freely and engaging in activities that had previously felt unattainable. Similar patterns have been described in qualitative research on orthognathic surgery, where patients reported increased self-awareness prior to surgery and predominantly positive reflections postoperatively (Cadogan & Bennun, 2011).

The findings suggest that orthognathic surgery within CLP care should be understood not solely as a functional and aesthetic intervention, but as a biographical transition with potential implications for identity and social participation (Bury, 1982). Clinical assessment may therefore benefit from explicitly addressing patients’ expectations, prior experiences of stigma, and personal meaning attributed to jaw discrepancy, as these factors have been shown to influence psychosocial adjustment in individuals with visible differences (Rumsey & Harcourt, 2004). Rather than assuming uniform psychosocial benefit, individualised dialogue regarding anticipated changes in self-perception and social interactions may support more realistic preparation.

Based on the findings, clinical practice may benefit from:

(1) structured preoperative discussions addressing expectations and psychosocial concerns,
(2) access to psychological support during the postoperative adjustment period, and
(3) opportunities for peer support to facilitate normalisation and shared understanding.

Future studies should explore the perspectives of individuals who decline orthognathic surgery in order to better understand the full spectrum of treatment decision-making and its psychosocial implications.

In conclusion, orthognathic surgery may, for some individuals with CLP, contribute to processes of psychosocial renegotiation characterised by increased confidence and social agency. However, these outcomes were neither universal nor automatic. The findings underscore the importance of individualised assessment and attention to psychosocial context throughout the surgical trajectory.

## Methodological considerations

Trustworthiness in qualitative research is commonly addressed through the criteria of credibility, transferability, dependability, and confirmability.

In the present study, credibility was strengthened by capturing participants’ experiences in their own words through verbatim quotations and by engaging multiple researchers in the analytic process. Reflexive discussions among the researchers allowed emerging interpretations to be critically examined and refined, thereby enhancing depth and interpretive sensitivity. No member checking was conducted. Although the absence of member checking may be considered a limitation, the interpretive approach and ongoing methodological debate regarding participant validation led us to support credibility instead through collaborative analysis, reflexive discussion and grounding interpretations in the empirical data.

Transferability was supported through detailed descriptions of the study context, participants and their pre-surgical situations, enabling readers to assess the relevance of the findings in other settings. The sampling strategy was further guided by the concept of information power, emphasising data richness and relevance in relation to the study aim.

Dependability was ensured by applying a transparent and systematic analytical approach, with clear documentation of methodological and analytical decisions throughout the research process. Confirmability was strengthened through ongoing discussions of interpretations within the research team and with colleagues, supporting reflexive awareness and reducing the influence of individual preconceptions on the findings.

The time interval between surgery and interview varied considerably among the participants, which may have influenced the accuracy and completeness of recollections. However, orthognathic surgery is commonly experienced as a highly significant and emotionally impactful life event. Previous research suggests that experiences associated with strong emotional engagement tend to be remembered with particular clarity and persistence (Dolcos et al., 2005; Qasim et al., 2023). From a qualitative perspective, the focus of this study was not to ensure exact factual recall, but on how the participants made sense of, and attributed meaning to their experiences over time. Temporal distance may thus have facilitated reflection and integration of the surgical experience into a broader life narrative, aligning with the study’s interpretive aim.

The heterogeneity in surgical techniques (CO and DO) and postoperative interval (0.3–19.8 years) may constitute a limitation in studies aiming at comparative outcome analysis, as different procedures and recovery trajectories may influence psychosocial experiences. Given the interpretative focus on meaning-making, rather than comparison, this variation was treated as part of the experiential context rather than as a variable for subgroup analysis. Nevertheless, future studies employing more homogeneous samples may further clarify technique-specific experiences.

Although empowerment emerged as an overarching theme, the analytic process actively sought disconfirming cases and variations, including neutral and ambivalent experiences, in order to avoid confirmatory bias. We also acknowledge that positive narratives were more prevalent in this material, which may reflect the composition of participants rather than analytic exclusion of less favourable accounts.

Participants who agreed to participate may represent individuals more willing to reflect on their experiences, which may have influenced the range of perspectives captured.

## Data Availability

The datasets generated and analysed during the current study are not publicly available due to confidentiality issues but are available from the corresponding author on reasonable request.

## Appendix I. Interview guide

**Introduction**

- Has the information sheet been reviewed and the informed consent form signed?
- Brief description of the purpose of the interview and the methodological approach.
- Explanation of why the participant has been invited to take part.
- Information that the interview will be audio-recorded and that participation is voluntary and may be discontinued at any time without consequences.

**Aim of the study**

The aim of the study is to illuminate the lived experiences of individuals with cleft lip and palate who have undergone orthognathic surgery.

**Can you tell me what made you want to have your jaw operated on?**

- Follow-up questions regarding function and/or appearance.
- Whose decision was it to undergo surgery?

**Can you describe how it was decided that you would undergo surgery?**

How did you experience the time before surgery/the waiting period?

**Can you describe what you thought and felt when you were informed that you would have surgery?**

- Can you tell me more about what made you feel that way?

**Can you tell me about the time when you had surgery?**

- How did you prepare for surgery?
- How did you experience the anaesthesia?
- How was the evening before surgery?
- How did you sleep the night before surgery?
- Do you remember waking up after surgery? How did you experience that?

**Can you tell me about the time after surgery? What was it like for you?**

- Did you have any positive experiences during the postoperative period?
- Did you have any negative experiences?
  ∘ How did you handle those experiences?

- Were there any parts of the treatment that you were responsible for managing yourself? (e.g., elastics, distractor)?
  ∘ How did you experience managing that responsibility?
  ∘ How did it feel when you were told that the jaw had reached a satisfactory position and that you could stop adjusting the distractor?

**How did your family and friends react after surgery?**

- How was it for you that they reacted in that way?
- Did you receive support from friends, family or others?
  ∘ What kind of support would you have needed?

**How were your school activities or work affected by the surgery?**

**What has the treatment meant for you in your life?**

- What has been positive?
  ∘ How have any positive effects influenced you?

- What has been negative?
  ∘ How have any negative effects influenced you?

**How did you experience the support from the healthcare staff during your treatment?**

- What could have improved your experience of the support?

**Do you have any advice for healthcare professionals regarding the treatment of patients who require orthognathic surgery?**

**Is there anything we have not discussed that you consider important in this context?**
